# The effect of chlorhexidine on dental calculus formation: an in vitro study

**DOI:** 10.1186/s12903-018-0517-3

**Published:** 2018-03-27

**Authors:** Yuuki Sakaue, Shoji Takenaka, Tatsuya Ohsumi, Hisanori Domon, Yutaka Terao, Yuichiro Noiri

**Affiliations:** 1 Division of Cariology, Operative Dentistry and Endodontics, Niigata University Graduate School of Medical and Dental Sciences, 2-5274, Gakkocho-dori, Chuo-ku, Niigata, 951-8514 Japan; 2 Division of Microbiology and Infectious Diseases, Niigata University Graduate School of Medical and Dental Sciences, 2-5274, Gakkocho-dori, Chuo-ku, Niigata, 951-8514 Japan

**Keywords:** Chlorhexidine, Oral biofilm, Dental calculus, Mineral uptake, SEM, EPMA

## Abstract

**Background:**

Chlorhexidine gluconate (CHG) has been proven to be effective in preventing and controlling biofilm formation. At the same time, an increase in calculus formation is known as one of considerable side effects. The purpose of this study was to investigate whether mineral deposition preceding a calculus formation would occur at an early stage after the use of CHG using an in vitro saliva-related biofilm model.

**Methods:**

Biofilms were developed on the MBEC™ device in brain heart infusion (BHI) broth containing 0.5% sucrose at 37 °C for 3 days under anaerobic conditions. Biofilms were periodically exposed to 1 min applications of 0.12% CHG every 12 h and incubated for up to 2 days in BHI containing a calcifying solution. Calcium and phosphate in the biofilm were measured using atomic absorption spectrophotometry and a phosphate assay kit, respectively. Morphological structure was observed using a scanning electron microscope (SEM), and chemical composition was analyzed with an electron probe microanalyzer (EPMA).

**Results:**

The concentrations of Ca and Pi following a single exposure to CHG increased significantly compared with the control. Repeatedly exposing biofilms to CHG dose-dependently increased Ca deposition, and the amount of Ca was five times as much as that of the control. Pi levels in CHG-treated biofilms were significantly higher than those from the control group (*p* < 0.05); however, the influence of the number of exposures was limited. Analyses using an SEM and EPMA showed many clusters containing calcium and phosphate complexes in CHG-treated biofilms. Upon composition analysis of the clusters, calcium was detected at a greater concentration than phosphate.

**Conclusions:**

Findings suggested that CHG may promote mineral uptake into the biofilm soon after its use. It is necessary to disrupt the biofilm prior to the start of a CHG mouthwash in order to reduce the side effects associated with this procedure. The management of patients is also important.

## Background

Periodontal diseases are initiated by bacterial biofilms that induce a host inflammatory immune response, which could lead to tooth loss and contribute to systemic inflammation [1]. Since the oral biofilm can be removed without surgical intervention, mechanical elimination such as brushing and flossing is fundamental for its control [2–4]. A chemical approach is used as an alternative or adjunctive method when elimination using dental instruments proves difficult. It has been demonstrated that adjunctive antimicrobials improve clinical parameters including plaque and gingival indexes by interfering with metabolic activities [2, 5, 6].

Chlorhexidine, a cationic bisbiguanide, is an antimicrobial agent with a broad spectrum of activity encompassing Gram-positive and Gram-negative yeasts, bacteria, dermatophytes, and some lipophilic viruses [7]. One of the most widely used and thoroughly investigated antiseptics is chlorhexidine gluconate (CHG), which is used in CHG oral rinse. This has been proven to be safe, stable, and effective in preventing plaque formation and inhibiting the development of gingivitis [7, 8].

It is well known that CHG causes considerable side effects, such as extrinsic staining, an alteration in taste perception, and an increase in calculus formation [9–12]. The calculus surface itself may not induce inflammation in the adjacent periodontal tissue [13, 14]. However, calculus formation is known to be a factor in plaque retention as well as a reservoir for toxic bacterial products and antigens [13]. In addition, a recent investigation has reported that treatment of *Porphyromonas gingivalis* biofilms with CHG for 5 min did not degrade their external structure or reduce the volume of protein and carbohydrate constituents [15]. The residual structure following CHG exposure may accelerate calculus formation and may serve as an ideal substrate to promote new microbial adhesion.

Although some clinical studies have demonstrated that CHG promotes calculus formation [8–11], the mechanism for the uptake of calcium and phosphate is unclear. The purpose of the present study was to examine the influence of CHG on calculus formation using an in vitro saliva-related plaque mineralization model. In particular, we investigated whether exposing a biofilm mass to CHG for a short period of time promoted the uptake of calcium and phosphate.

## Methods

### Saliva collection and preparation

Human saliva that had been stimulated by chewing wax was collected from one healthy male (one of the authors) who had not consumed food for 2 h prior to donation. The subject had no evidence of present caries and had not taken antibiotics for at least 3 months prior to donation. The saliva was centrifuged at 10,000 *g* for 10 min, and the pellet was re-suspended in brain heart infusion (BHI) broth (Becton, Dickinson, and Company, Sparks, MD, USA). The suspension was adjusted to an OD_600_ of 0.2 and was used as an inoculum for biofilm growth. The supernatant was sterilized as described previously [16] and used for pellicle formation.

### Biofilm formation

**Fig. 1:**
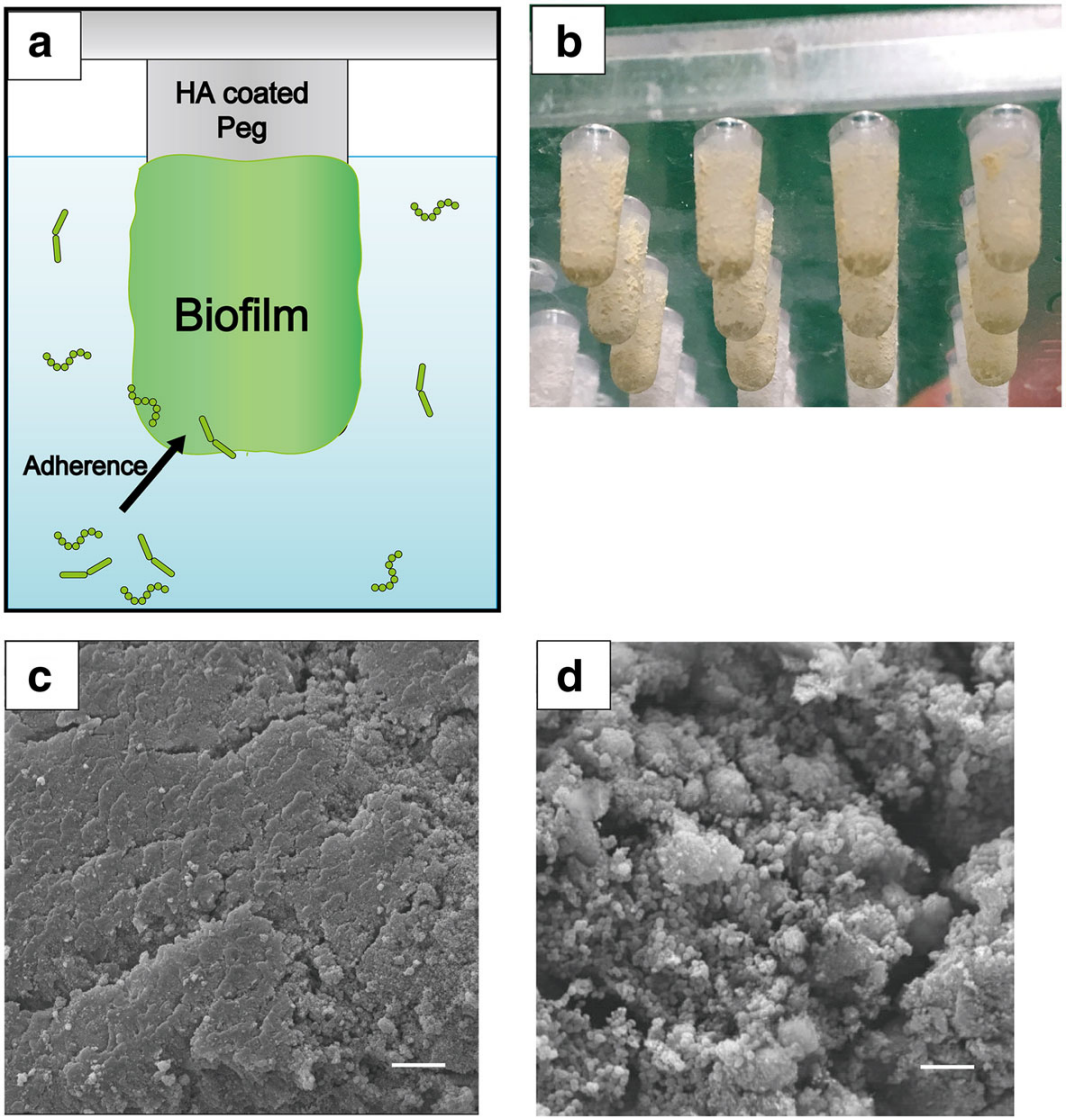
Biofilm formation using an MBEC™ device. **a** A representation of biofilm formation on a peg. **b** Biofilm formation on a peg after 3 days incubation. **c** An SEM image of a peg. **d** An SEM image of saliva-related biofilm developed on the peg after 3 days incubation. Bars = 5.0 μm

Biofilms were generated using an MBEC™ device (Innovotech, Edmonton, Canada) as per the method described by Pesciaroli et al. [17] with some modifications. The device consisted of a plastic lid with 96 hydroxyapatite (HA)-coated pegs and individual wells (Fig. 1). Following pellicle formation for 2 h at 37 °C, the pegs were transferred into 200 μl of the bacterial inoculum described above and incubated for 1 h at 37 °C under aerobic conditions, allowing the microorganisms to colonize the peg. The pegs were then transferred into BHI broth containing 0.5% sucrose and incubated under anaerobic conditions at 37 °C for 3 days. The medium was changed every 12 h until the day of harvesting.

### CHG treatment and mineral uptake

**Fig. 2:**
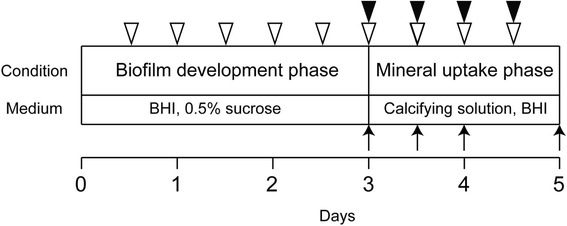
Experimental design showing time schedule, procedures, and sampling. White arrow heads indicate medium exchange, and black arrow heads indicate CHG exposure. Sampling and analysis were performed at the points indicated by black arrows

The biofilms that formed on the pegs were washed to remove unbound cells by placing the lid into the rinse plate with ion exchanged water (IEW) for 10 s. They were then exposed for 1 min to distilled water (DW) or 0.12% CHG (Sigma-Aldrich, St. Louis, MO, USA), which is the concentration involved in a conventional mouthwash product [18, 19]. After washing with IEW for 1 min, the biofilms were transferred into calcifying solution and incubated for up to 48 h at a maximum of 37 °C under anaerobic conditions (N_2_ 85%, CO_2_ 10%, O_2_ 5%). The calcifying solution was composed of Solution A (NaCl 2.1 g, KCL 1.56 g, Na_2_HPO_4_·12H_2_O 0.9 g, NaH_2_PO_4_· 2H_2_O 0.39 g, KSCN 0.87 g, Urea 0.2 g, 3,3-dimethylglutaric acid 0.8 g, NaOH 0.36 g, and BHI medium 900 ml) and Solution B (CaCl_2_·6H_2_O 0.33 g and distilled water 100 ml) [20]. Solutions A and B were sterilized separately for 20 min at 121 °C and mixed together just before use. Biofilms were exposed to CHG for 1 min every 12 h and transferred into a new calcifying solution. Treatment with DW served as the control. Biofilms were also immersed in BHI instead of the calcifying solution to determine the amount of mineral uptake from the media. A summary of the experimental design is displayed in Fig. 2. During the experimental process, bacterial viability was checked by assessing the turbidity of the media.

### Mineral analysis

Mineral analysis was performed using a modification of the method described by Wong and Sissons [21]. Following assigned treatment (0, 12, 24, and 48 h in mineral uptake phase; Fig. 2), biofilms were extracted with 1 ml of 0.5 mol/l perchloric acid (Nacalai Tesque, Inc., Kyoto, Japan) and centrifuged at 15,000 *g* for 15 min. Calcium (Ca) in the supernatant was measured by inductively coupled plasma-atomic emission spectrometry (SPS1500; Seiko Instruments, Inc., Tokyo, Japan), and phosphate (Pi) was measured using the Malachite Green Phosphate Assay Kit (Bioassay Systems, Hayward, CA, USA), according to the manufacturer’s instructions. The pellet was re-suspended in 1 ml of distilled water and the optical density (OD) at 620 nm was recorded. Calculated Ca and Pi concentrations (ppm) were converted to values that corresponded to the same bacterial volume (OD = 1.0) [22]. This assay was performed with a total of four replicates per treatment.

### Morphological and chemical composition analyses

Pegs were broken from the lid and placed into an empty receiver vial, and specimens were desiccated in a dry box. Thereafter, biofilms were fixed with 2% paraformaldehyde and 2.5% glutaraldehyde in 0.1 mol/l cacodylate buffer for 1 h at room temperature. All specimens were washed twice with phosphate buffered saline and dehydrated in a graded ethanol series (50, 60, 70, 80, 90, 95, and 100% for 10 min each). Specimens were then mounted on carbon stubs and sputter-coated with a 300-Å-thick gold layer using an ion coater (IC-50; Shimadzu, Kyoto, Japan). Morphology and elemental composition were analyzed using a wavelength-dispersive X-ray spectroscopy electron probe microanalyzer with an image observation function (SEM-EPMA, EPMA1601; Shimadzu). The limit of detection of the elements was approximately 1 μm in depth [23]. This assay was performed with a total of four replicates per treatment. For image analysis, three sample locations on the biofilm surface were randomly selected on each image, and the Ca: Pi ratio at each location was calculated.

### Statistical analysis

Statistical analysis was performed using SPSS 11.0 (SPSS Inc., Chicago, USA) and excel-toukei 7.0 (ESUMI Co., Ltd., Tokyo, Japan). Where applicable, data are presented as means ± standard deviation (SD). Statistical differences between the different groups were analyzed using a two-way factorial ANOVA without replication and the Steel-Dwass test. Differences were considered significant at *p* < 0.05.

## Results

### Effect of chlorhexidine on the deposition of ca and pi in biofilms

**Fig. 3:**
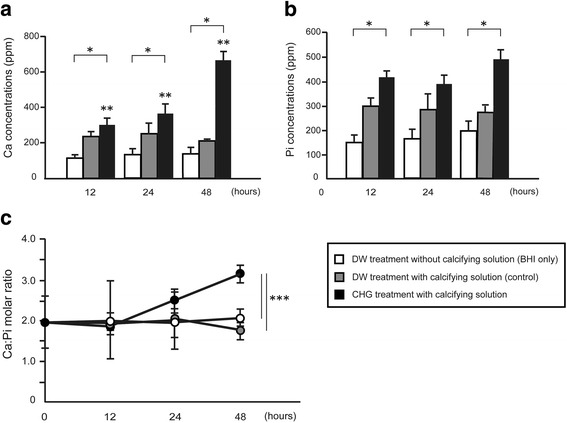
Ca and Pi uptake inside biofilm masses exposed to CHG. Ca (**a**) and Pi (**b**) concentrations and Ca: Pi (**c**) molar ratio in biofilms were determined. The three-day-old biofilms were periodically exposed to 1 min applications of 0.12% CHG every 12 h and incubated for up to 2 days in BHI containing a calcifying solution. Distilled water was used as the control. Results are shown as the mean ± SD of four independent experiments. There was a significant difference among three experimental conditions (indicated by bracket, *p < 0.05). Incubation time affected calcium deposition (**p < 0.05). There was a significant difference in Ca: Pi molar ratio after 48 h incubation between the CHG treatment group and the others (***p < 0.05)

The calcifying solution promoted the uptake of both Ca and Pi in biofilms regardless of CHG exposure (Fig. 3a and b). The prolonged incubation time alone, without CHG exposure, did not influence mineral uptake. Incubation for 12 h following a single exposure to CHG significantly increased the amount of minerals in biofilms compared with the control (*p* < 0.05). Repeatedly exposing biofilms to CHG dose-dependently increased Ca deposition, and the amount of Ca was five times as much as that of the control (Fig. 3a). Pi levels in CHG-treated biofilms were significantly higher than those from the control group (*p* < 0.05); however, the number of exposures did not influence results significantly (Fig. 3b, *p* > 0.05). Although the Ca: Pi molar ratio of the total mineral content in CHG-treated biofilms was almost the same as control biofilms between 0 and 24 h, a significant increase was observed in CHG-treated biofilms at 48 h (Fig. 3c, *p* < 0.05).

### Morphological and chemical composition analysis

**Fig. 4:**
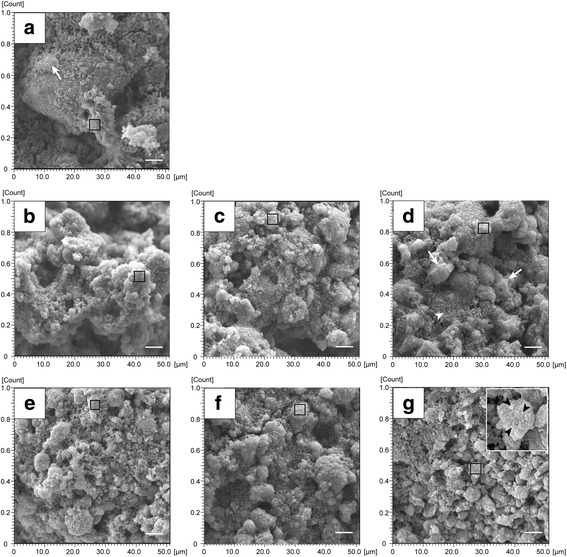
Representative SEM images in the mineral uptake phase. Figure shows DW treatment with calcifying solution for 0, 12, 24, and 48 h (**a**–**d**) and CHG treatment with calcifying solution for 12, 24, and 48 h (**e**–**g**). White arrow heads indicate microorganisms, and white arrows show EPS-like structures. Calcium phosphate-like particles were seen, especially on the surface of the biofilm after 48 h incubation (inset in g; higher magnification of the area indicated by a rectangle). Scale bars = 5.0 μm

An SEM-EPMA enabled morphological observation and quantitative elemental analyses of Ca and Pi from the same field of view. We have presented representative images in Fig. 4. Analysis of SEM data showed that cocci- and bacilli-like bacteria were densely packed within extracellular polysaccharide (EPS)-like structures on the surface of pegs after 3 days of incubation (Fig. 4a). In the control group (Fig. 4b-d), biofilms developed three-dimensionally during further incubation for two days. Microorganisms (white arrow head) were observed to be embedded in EPS-like structures (white arrow). In the CHG treatment group, biofilm structures remained on the peg even after repeated exposure to CHG (Fig. 4e-g). Biofilms in the shape of clusters were observed on the surface, and these clusters were relatively small in comparison with the control. Bacterial growth was achieved in the media until 24 h incubation following exposure to CHG, and it was not detected after 36 h, meaning that microorganisms in the biofilm were completely disinfected after 48 h. Many small particles, which were not microorganisms, were observed on the surface of the biofilm, especially in the CHG treatment group (black arrow heads in the inset of Fig. 4g).

**Fig. 5:**
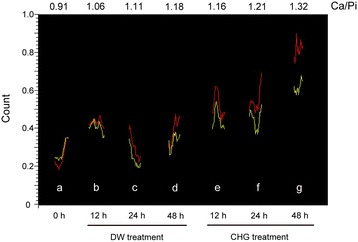
Profiles of Ca and Pi within a region determined by a rectangle in Fig. 4 The red line graph indicates Ca, and the yellow line graph indicates Pi. The Y axis represents the count, which indicates X-ray intensity of the spectral peak. Scale bars = 5.0 μm

We performed EPMA analysis to determine whether these particles were of mineral composition. Figure 5 showed the profiles of Ca and Pi within a region determined by a rectangle in Fig. 4. The red line graph indicates Ca, and the yellow line graph indicates Pi. The Y axis represents the count, which indicates the X-ray intensity of the spectral peak. The mean Ca: Pi ratio in the control group was 1.06 ± 0.12, 1.12 ± 0.18, and 1.16 ± 0.22 (mean ± standard deviation) for 12, 24, and 48 h incubation, respectively. The mean Ca: Pi ratio in the CHG treatment group was 1.18 ± 0.18, 1.20 ± 0.20, and 1.40 ± 0.26 (mean ± standard deviation) for 12, 24, and 48 h incubation, respectively. There was a significant difference between the control group and the CHG treatment group after incubation for 48 h (*p* < 0.05).

## Discussion

Numerous clinical studies for 6 months have demonstrated that the use of antimicrobial mouthwashes such as CHG as part of daily oral care can reduce plaque and gingivitis [8, 11, 24]. However, rinsing with CHG for 4 weeks or longer causes considerable side effects, such as calculus build up, extrinsic tooth staining, transient taste disturbance, and effects on the oral mucosa [11, 25].

In this study, we investigated whether mineral deposition preceding calculus formation would occur at an early stage using saliva-related biofilms. Our results showed that mineral uptake inside a CHG-treated biofilm significantly increased compared with the control after 48 h (four exposures) in the presence of calcifying solution (Fig. 3). In SEM and EPMA analyses, small apatite-like particles that contained more Ca than Pi were observed in CHG-treated biofilms at 48 h (Figs. 4g and 5).

Although plaque hardening caused by the precipitation of mineral salts usually begins between Day 1 and Day 14 of plaque formation, mineralization has been reported to occur as soon as 4–8 h [7]. Eilberg et al. [22] tested the mineralizing activities of plaque using samples from humans. When plaque samples were placed in calcifying solution for 24 h, the amounts of mineral they contained ranged from 0.37 to 50 μg for Ca/OD Unit, 0.11 to 21 μg for Pi/OD Unit, and 1.02 to 5.6 for Ca: Pi ratio. Previous and present findings suggest that dental plaque might absorb minerals from the oral environment, and CHG might promote its deposition.

Dental calculi are reported to contain the following four calcium phosphate compounds: hydroxyapatite, whitlockite, octacalcium phosphate, and brushite with Ca: Pi ratios of 1.67, 1.5, 1.33, and 1.0, respectively [26]. When plaque mineralization begins, brushite develops into octacalcium phosphate, hydroxyapatite, and whitlockite [27]. In this study, the ratios of Ca and Pi in the experimental group were relatively higher than those in control group (Figs. 3, 4 and 5). In addition, a Ca-rich component (Ca: Pi = 1.32) was detected on the surface of CHG-treated biofilms at 48 h (Fig. 5g). It is possible that CHG may favor its calcification.

This is the first report demonstrating the acceleration of mineral uptake into biofilms caused by in vitro CHG exposure. Although the mechanism remains unclear, there are two possible explanations. Firstly, CHG is a cationic compound, and it is rapidly attracted to negatively-charged bacterial cell surfaces. This alters the integrity of the bacterial cell membrane and binds to phospholipids in the inner membrane, and the leakage of low-molecular-weight components follows [7]. However, the biofilm structure remains intact on an adhered site [15]. It is possible that denatured components on the biofilm surface may become crystallized nuclei that enlarge and coalesce to form a calcified mass. In addition, since calcium binds to lipoteichoic acid, the compromised surface may promote the deposition [28].

The other possibility is that pH in the biofilm may increase as a result of the antimicrobial effect of CHG. In this study, bacterial growth was not detected after 36 h, meaning that the microorganisms in the biofilm died. The fact that the pH was neutral between 24 and 48 h may have aided calcification. It has been reported that an alkaline pH in biofilms is critical for the promotion of plaque mineralization [20, 29]. In fact, calcium uptake significantly increased after 48 h (four exposures) (Fig. 3).

Calculus formation is not the main etiological factor. Jepsen et al. stated that periodontal healing occurs even in the presence of calculi, as long as bacteria are removed or disinfected [13]. For example, it has been reported that an autoclaved calculus does not cause pronounced inflammation or the formation of abscesses in connective tissues [30]. However, calculus formation is a secondary etiological factor. A report of histological sections of a human tooth root showed that calculi were covered with viable bacterial plaque [13]. Nichols et al. reported that the dihydroceramide lipids produced by *P. gingivalis* were found in a subgingival calculus [31]. Thus, it is critical to prevent calculus formation. Although CHG mouthrinse has been proven to be effective for inhibiting gingivitis, the management of patients is important, because mineral uptake into the biofilm occurs in the early stage.

## Conclusions

Within the limitations of the present study, CHG may promote mineral uptake into the biofilm soon after the first application. It is necessary to disrupt the biofilm prior to the start of CHG mouthwashes in order to reduce side effects, and the management of patients is also important.

## Abbreviations

BHI: Brain heart infusion
Ca: Calcium
CHG: Chlorhexidine gluconate
DW: Distilled water
EPMA: Electron probe microanalyzer
EPS: Extracellular polysaccharide
HA: Hydroxyapatite
IEW: Ion exchanged water
Pi: Phosphate
SEM: Scanning electron microscope
